# Medical Society of the State of Pennsylvania

**Published:** 1855-07

**Authors:** D. Francis Condie, Henry Carpenter

**Affiliations:** Secretary; Secretary


### MEDICAL SOCIETY OF THE STATE OF PENNSYLVANIA.

This Society held its annual meeting at Hollidaysburg, on Wednesday and Thursday, May 30 and 31.

The meeting was called to order at 10 o'clock, May 30, by the President, Dr. Gemmill.

Drs. G. Emerson, J. A. Landis, and J. McCulloch, were appointed a committee to examine and report on the credentials of members.

This committee reported the names of those entitled to seats.

On motion of Dr. A. S. Kennedy, Dr. R. P. Thomas, of Philadelphia, was appointed Treasurer, *pro tempore*, in the absence of the Treasurer, Dr. West.

The Committee on Credentials report that since the last session two new societies had been organized, viz: one for Franklin, and one for Washington County; the constitutions and by laws of both of which had been approved by the Censors of their respective Districts.

On motion of Dr. H. Carpenter, the reading of the minutes of the last session of the Society was dispensed with.

The session was formally opened by the President, Dr. Gemmill, with a brief but pertinent address on the importance of the complete and systematic organization of the medical profession throughout the State, and the leading objects to be gained by such organization.

On motion of Dr. F. G. Smith, the thanks of the Society were tendered to Dr. Gemmill for his able and eloquent address, and that he be requested to furnish a copy for insertion in the printed *Transactions.*

On motion of Dr. Morton, it was

*Resolved*, that the sessions of the Society be from nine to twelve o'clock A. M., and from two to five P. M.

On motion of Dr. Ross, it was

*Resolved*, That the reporters of the public press have every facility afforded them for taking minutes of the proceedings.

On motion of Dr. Thomas, it was

*Resolved*, That a Committee of Nomination, to consist of one from each County represented, selected by its delegates in attendance, be appointed ; and that said committee be instructed to designate the place of meeting of this Society in 1856.

The report of the *Chester County Medical Society* was presented and read, and, on motion, referred to the Committee of Publication. It being understood that the committee by whom the report was drawn up have the privilege of making such corrections therein as they may deem proper.

Afternoon Session, *May* 30, 1855.

The Minutes of the morning session were read and approved.

The Committee of Publication presented their report, as follows:—

The Committee of Publication beg leave to report that they have had printed *seven hundred and fifty* copies of the *Transactions* of the session of 1854, at an expense of $316.45.

Copies were transmitted to the several County Societies, in proportion, as far as it could be effected, to their respective ratio of representation.

The Treasurer, Dr. F. West, presented his report, showing a balance in the Treasury of $32.

On motion of Dr. Condie, it was

*Resolved*, That the account of the Treasurer, with the vouchers accompanying the same, be referred to an auditing committee, and Drs. S. Lewis, S. A. Ogier, and J. H. Seltzer, were appointed the committee.

The following gentlemen were announced as constituting the Nominating Committee, agreeably to the resolution adopted at the morning session:—

*Berks*—Dr. J. H. Seltzer.
*Blair*—Dr. J. A. Landis.
*Cambria*—Dr. W. A. Smith.
*Chester*—Dr. S. A. Ogier.
*Delaware*—Dr. C. J. Morton.
*Huntingdon*—Dr. H. Orlady.
*Lancaster*—Dr. J. H. Ehler.
*Mifflin*—Dr. Bowers.
*Montgomery*—Dr. F. B. Poley.

*Philadelphia*—Dr. S. L. Hollingsworth.
*Schuylkill*—Dr. J. S. Carpenter.
*Washington*—Dr. J. Wishart.

The report of the *Huntingdon County Medical Society* was presented and read, and, on motion, referred to the Committee of Publication.

The report of the *Schuylkill County Medical Society* was presented and read, and, on motion, referred to the Committee of Publication.

On motion of Dr. Yardley, it was

*Resolved*, that a committee be appointed to examine the minutes of last session, and report any unfinished business that may appear thereon requiring action at the present session.

Drs. Yardley, Shrack, and Christie were appointed the committee.

A communication was received from the Reading-Room Association, of Hollidaysburg, granting the use of their reading-room to the Delegates to the Pennsylvania State Society during their sojourn in the borough.

On motion of Dr. Condie, the privilege thus granted was very gratefully received, and the thanks of the Society were directed to be presented to the association for their very polite and liberal action in the premises.

The report of the *Medical Society of the City of Reading and County of Berks* was presented and read, and, on motion, referred to the Committee of Publication.

On motion of Dr. Carpenter, of Lancaster, the resolutions in reference to delinquent members appended to said report, were laid upon the table.

The Committee on Unfinished Business in the minutes of last session, presented the following report:—

The Committee on Unfinished Business report that they have examined the minutes of last years' proceedings, and find the following committees were continued to report at this session, namely,

1st. Committee on Adulteration of Drugs. (See printed *Transactions*, vol. iv. p. 10.)

2d. Committee on Vaccination and the Preservation of Vaccine Virus. (See printed *Transactions*, vol. iv. p. 10.)

They further find that reports are due from the following committees appointed at the last session, namely,

1st. Committee on Blanks for Reports from County Societies. (See printed *Transactions*, vol. iv. p. 13.)

2d. Committee on Resolutions submitted by the Chester County Medical Society, through Dr. Parrish. (See printed *Transactions*, vol. iv. p. 17.)

Signed,

Thos. H. Yardley,

John Shrack,

R. W. Christie.

On motion of Dr. Condie, it was

*Resolved*, That the several items of business referred to in said report, be now taken up *seriatim*.

It was stated, on behalf of the Chairman of the Committee on Adulteration of Drugs, that he had prepared a report on the subject which would, no doubt, be presented in the course of the present session.

On motion, the committee was continued.

Dr. Carpenter, of Lancaster, stated that he had been requested by Dr. Parry, the Chairman of the Committee on Vaccination, &c., to state that, inasmuch as no few facts had reached the committee in reference to the subject committed to them, they had not thought it worth while to prepare a report from the materials they had been enabled to collect, and begged to be discharged from the further consideration of the subject.

Dr. Condie remarked, that, inasmuch as he was convinced that a digest of the facts at present known in reference to the preservative powers of vaccination, and to the preservation of vaccine virus in a state best adapted to produce effectually its influence upon the system of those in whom it is inserted, and a comparison of the experience of the medical profession in different portions of Europe on the several points involved in these important questions, as given in the reports of several medical associations abroad, would be acceptable to the great body of the profession throughout our State, and that it would be calculated also to remove some erroneous views and practices still entertained, and pursued in relation to vaccination, and, at the same time, to promote the extension of vaccination among the community at large, by rendering its protective powers more uniform and certain; he therefore, moved that the committee be continued, with instructions to report at the next session of the Society. Which resolution was unanimously adopted.

Dr. Cassidy asked leave to withdraw from the committee, which request was not granted.

The Chairman of the Committee on Blanks for Reports of County Societies presented his report, which was laid upon the table for the present.

The Chairman of the Committee on Resolutions, presented by the Chester County Medical Society, not being present, the Committee was, on motion, continued with instructions to report at the next session.

The committee appointed to audit the Treasurer's accounts, reported as follows:—

The committee appointed to audit the Treasurer's account, beg leave to report that they have attended to that duty, and find the said accounts correct. A balance remains in his hands of thirty-two dollars ($32.) There is a balance due by the Society for printing the *Transactions* for 1854, amounting to eighty-seven dollars thirty-two cents ($87.32.) Assessments are still due from the societies of Erie, Lycoming. Mercer, and York, for 1853 and 1854; and from Alleghany, Bradford, Cambria, Lebanon, Perry, and Susquehanna, for 1854, amounting in all to one hundred and fifty-seven dollars ($157.)

Signed, SAMUEL LEWIS,
J. H. SELTZER,
SEPT. A. OGIER.

The following amendment to the Constitution was presented by Dr. Yardley: To insert in Sect. 2, Article III., after the word "societies," in the second line, the words "provided, however, that the officers of the society, and the chairmen of all committees who have reports to present, shall be delegates *ex-officio.*" So that the section shall read:

"SECT. 2. The delegates shall receive their appointments from the county societies; provided, however, that the officers of the societies, and the chairmen of all committees who have reports to present, shall be delegates *ex-officio;* and to them shall be intrusted the management of the business and affairs of the Society."

The said amendment was unanimously adopted.

On motion of Dr. Kennedy, the following resolution was adopted:—

*Resolved,* That the Corresponding Secretary be, and he is hereby instructed to address a circular to the prominent physicians in those portions of the State in which no county medical societies exist, urging upon them the importance of organizing such societies without delay.

The report of the Committee on Forms of Reports for County Societies was presented and approved.

The resolution appended to said report was adopted, namely,

"*Resolved,* That the Committee on Forms for County Reports be, and they are hereby instructed to have printed a sufficient number of the forms as reported, and to transmit the same to the secretaries of the county societies, with the request that returns be made at the next session of the State Society in accordance therewith."

Dr. Morton offered the following preamble and resolution:—

*Whereas,* The editors of the *Delaware County Republican, Blair County Whig, Raftsman's Journal,* and *Olive Branch,* have, with a spirit of genuine philanthropy, constantly refused to insert, in their journals, the advertisements of patent or quack nostrums, and

*Whereas,* They have been laboring with a faithful and unapplauded zeal, to enlighten the public mind as to their injurious tendencies, thereby enabling the community to detect and discard the ignorant and illiterate pretender, who has too generally been successful, with the aid of a well-paid press, to foist upon the ignorant and credulous his vile drugs; thus jeopardizing the health and lives of the people, both by their indiscriminate use and by contravening suitable and efficient medication: Therefore, as an evidence of our appreciation of a course so laudable and self-sacrificing, the Medical Society of the State of Pennsylvania, do

*Resolve,* That the members of the County Medical Societies be requested to extend to them, as well as to their families while under their charge, the gratuitous services of the medical profession; and they further

*Resolve,* That the County Societies be requested to append to their annual reports the names of all editors, and of their respective journals, who have constantly refused to insert the advertisements of empirical nostrums, and that the Committee of Publication be hereby authorized to furnish them annually with a copy of the *Transactions* of the Society.

On motion of Dr. Hartshorne, the said preamble and resolutions were referred to a committee of three, to report to-morrow morning.

Committee—Drs. Hartshorne, Morton, and Orlady.

The Committee of Nominations presented a report, which, on motion of Drs. Condie, and H. Carpenter, was referred back to the Committee, with instruction that it is the sense of this Society that, agreeably to the true meaning of the Constitution, no one can be placed on nomination for the offices of President, Vice-Presidents, Secretaries, Treasurer, or Delegates to the American Medical Association excepting Delegates who are in actual attendance at the sessions of the Society.

[The Report of the Committee of Nominations was referred back, to them, as it contained the names of three gentlemen who were deemed constitutionally ineligible to office, from the circumstance that they were either not delegates, or being delegates, were absent from the meeting. One of the gentlemen referred to was not a delegate. One had been elected a delegate, but had resigned, and one was a delegate, who, though not present when the report was read, took his seat before the final action of the Society upon the matter. In the discussion which followed the reading of the report, it was argued that if the precedent of electing persons to office who were not delegates, or who, if delegates, were not present, were adopted, it would prove injurious if not fatal, to the best interests of the Society. On those grounds, and on that of unconstitutionality, the Committee was ordered to replace the objectionable names by others of delegates in attendance. We do not consider that the Committee is much to be blamed in the matter, however, as their action was only in accordance with that of every previous Committee of Nominations since the organization of the Society, as the following statement amply shows:

In 1849, Dr. Isaac Hays, not a delegate, was elected Corresponding Secretary.
" " Dr. Geo. B. Kerfoot, not a delegate, was elected Recording Secretary.
" " Dr. Thos. Wood, not a delegate, was elected a delegate to the American Medical Association.
" 1850, Dr. J. T. Allen, not a delegate, was elected a Vice President.
" " Dr. P. F. Nagle, not present, was elected a delegate to the American Medical Association.
" 1851, Dr. Geo. Fox, a delegate, but not present, was elected Treasurer.
" " Dr. Traill Green, not a delegate, was elected a delegate to the American Medical Association.
" " Dr. J. L. Foulke, not a delegate, was elected a delegate to the American Medical Association.
" 1852, Dr. Joseph Gibbons, a delegate, but not present, was elected Rec. Sec.
" " Dr. F. S. Burrowes, " " " " a delegate to the American Medical Association.
" " Dr. R. E. James, not a delegate, was elected a delegate to the A. M. Asso.
" " Dr. Jas. R. McCoy, " " " " "
" 1853, Dr. Traill Green, " " " " "
" " Dr. Edw. Wallace, " " " " "
" " Dr. John S. Ross, " " " " "
" " Dr. John S. Irvine, " " " " "
" 1854, Dr. Jacob M. Gemmill, a delegate, but not present, was elected Presid't.
" " Dr. J. H. Dorsey, a delegate, but not present, was elected a delegate to the American Medical Association.
" " Dr. Joseph Henderson, not a delegate, was elected a delegate to the American Medical Association.

No happier illustration than the above can be given of the sharpening influence of the mountain air upon the intellect; it is clear that the constitution was never understood until the meeting at Hollidaysburg.—Ed.]

The report of the *Philadelphia County Medical Society* was presented and read, and, on motion, it was referred to the Committee of Publication.

The report of the *Lancaster County Medical Society* was presented and read, and, on motion, it was referred to the Committee of Publication.

Morning Session, *May* 31, 1855.

The minutes of yesterday afternoon's session were read and approved.

The Committee on preamble and resolutions of Dr. Morton, presented the following report:—

The Committee appointed to consider the preamble and resolutions offered by Dr. C. J. Morton, in regard to the action of certain editors of the public press, in declining the insertion of quack advertisements, report that they are prepared to recommend the adoption of the preamble, with the alteration of a few words only, and that they would respectfully suggest the substitution, instead of the resolutions in question, of a single resolution, expressive of the sentiment of the Society, so that the preamble and resolution, as thus modified, may, together, read as follows:—

*Whereas*, The editors of the *Delaware County Republican*, *Blair County Whig*, and those of some other papers in this State, have, with a spirit of genuine philanthropy, and at considerable sacrifice, constantly refused to insert, in their journals, the advertisements of patent or quack nostrums, and

*Whereas*, They have zealously labored to enlighten the public mind on the injurious tendency of such impositions, and, by withholding from them the influence of the press, to lessen the power of pretenders to jeopardize the health and lives of the ignorant and credulous, through the indiscriminate use of their compounds and the contravention by such use of suitable and efficient medication; therefore,

*Resolved*, That this Society views with high approbation and satisfaction the course of the editors and publishers alluded to, as furnishing an example well worthy to be followed by all who conduct the public press, and that we recommend to those members of the profession who reside within the vicinity of such journals, to afford them their patronage, and to testify, in such mode as their own sense of propriety and courtesy may dictate, their opinion of the obligation due from us to those who thus sustain the cause of legitimate medicine.

Signed, Henry Hartshorne,
Chas. J. Morton,
H. Orlady.

On motion of Dr. Carpenter, of Schuylkill, the report was adopted.

Dr. Carpenter, of Schuylkill, offered the following resolution:—

*Resolved*, That the members of this Society; and the profession generally, be strongly advised to use their influence with apothecaries and druggists to induce them to discontinue the purchase and sale of patent and quack medicines. And that the profession be recommended to furnish their patronage to those only who comply in this respect.

Dr. Neill moved to amend by adding after "quack medicines" the words "and patented instruments."

The proposed amendment gave rise to considerable discussion. Dr. Hartshorne moved to lay the whole subject upon the table, which was not agreed to. Finally, on motion of Dr. Biddle, the whole subject was referred to a committee of three—Drs. Condie of Philadelphia, Carpenter of Schuylkill, and Ogier of Chester—with instructions to report at the next session of the Society.

The following report was presented:—

The special committee, to whom were referred certain resolutions from the Berks County Medical Society, on the subject of the adulteration of drugs manufactured in the United States, have the honor to submit the following report:—

That, in their opinion, the suggestions of the Berks County Medical Society are worthy the consideration and endorsement of the State Medical Society. The committee believe that the appointment of an *analytical commission,* to investigate the quality of drugs manufactured in the United States, under the sanction of the State Medical Society, might be productive of eminent benefit.

They recommend, therefore, the following resolution:—

*Resolved,* That an analytical commission of three members be appointed by the State Medical Society, to investigate the quality of drugs manufactured in the United States. That this commission shall report within two years to the Society, and that the necessary expenses of said investigation shall be paid by the Society.

Signed, J. B. Biddle,
J. Carson.

The report was adopted, the resolution thereunto appended adopted, and Drs. Biddle, Carson, and Lewis appointed the commission thereby created.

Dr. Thomas offered the following preamble and resolution, which were unanimously adopted.

*Whereas,* Section 1, of Article IV. of the Constitution, provides for the election of five Censors for each of the six censorial districts, and Sects. 6 and 7 of Art. V. constitute the Censors of each district a court of final jurisdiction in cases of appeal from the decisions of county societies; and, whereas, in the present organization of this Society it is impossible to make appointments for each of the six districts, therefore,

*Resolved,* That the Censors elected conjointly for the first and second districts, for the third and fourth districts, and for the fifth and sixth districts, shall have full and complete jurisdiction, under the Constitution, in all cases arising, irrespectively, in the first and second, in the third and fourth, and in the fifth and sixth districts.

The report of the *Blair County Medical Society* was presented and read, and, on motion, referred to the Committee of Publication.

The report of the *Delaware County Medical Society* was presented and read, and, on motion, referred to the Committee of Publication.

Lists of the officers and members of Alleghany, Washington, and Franklin County Medical Societies were presented.

On motion of Dr. Kennedy, it was

*Resolved*, That the Recording Secretaries be instructed to prepare a digested index to the Reports of the County Societies for the last five years, for insertion in the forthcoming volume of *Transactions*.

On motion of Dr. Biddle, it was

*Resolved*, That a Special committee be appointed to report a plan for effecting an extension of the organization into medical societies of the regular members of the profession throughout the State; and that said committee be requested to take, forthwith, such measures as they may deem proper to effect such organization.

Committee—Drs. Gemmil, Biddle, and Wythes.

On motion of Dr. Condie, a committee, consisting of Drs. Condie and Saltzer, was appointed to draw up and present, at the next session of this Society, resolutions expressive of the feelings of its members on the loss they have sustained in the death of their late esteemed member and ex-President, Dr. John P. Heister, of Berks Co., and of their desire to commemorate his worth as a man and his talents and skill as a physician.

On motion of Dr. Henry Carpenter, a committee, consisting of Drs. H. Carpenter, and J. M. Gemmill, was appointed to present at the next session, resolutions expressive of the feelings of this Society on the loss they have sustained in the death of their late esteemed associate, Dr. C. O. Richards, of Lancaster.

On motion of Dr. Cassidy, the same committee were instructed to present, at the next session, resolutions of similar import in reference to the death of Dr. A. S. Baer, of Lancaster.

The amended report of the Committee of Nominations being read, on motion of Dr. Condie, it was

*Resolved*, That the officers therein named be, and they are hereby declared to be, the officers of this Society for the ensuing year.

*President.*—James S. Carpenter, of Schuylkill Co.

*Vice-Presidents.*—J. Wishart, of Washington County; P. Cassidy, of Lancaster do; G. Emerson, of Philadelphia do; J. Shrack, of Montgomery do.

*Recording Secretaries.*—H. Carpenter, of Lancaster County; A. L. Kennedy, of Philadelphia do.

*Corresponding Secretary.*—T. H. Yardley, of Philadelphia.

*Treasurer.*—R. P. Thomas, of Philadelphia.

*Censors, First and Second Districts.*—J. B. Biddle, Philadelphia County, J. L. Atlee, Sen., Lancaster do.; W. Worthington, Chester do.; Hiram Corson, Montgomery, do.; Chas. Martin, Lebanon, do.; R. E. James, Northampton do.; G. F. Horton, Bradford do.; Ed. Wallace, Berks do.

*Third and Fourth Districts.*—Jos. A. Landis, Blair County; J. B. Luden, Huntingdon do.; Thos. Wood, Lycoming do.; Jos. Henderson, Mifflin do.; J. H. Case, Perry do.

*Fifth and Sixth District.*—Jno. Wishart, of Washington County;

Addison, Pittsburg; J. P. Gazzam, Alleghany do.; J. T. Ray, Mercer do.; C. F. Perkins, Erie do.

*Delegates to American Medical Association.*—R. W. Christy, Blair County; J. H. Seltzer, Berks do.; F. B. Poley, Montgomery do.; C. J. Morton, Delaware do.; J. M. Gemmill, Huntingdon do.; Chas. Bower, Mifflin do.; John Wishart, Washington do.; J. S. Carpenter, Schuylkill do.; S. A. Ogier, Chester do.; A. Sheller, Lancaster do.; H. Orlady, Huntingdon do.

*Additional Members of the Committee of Publication.*—F. G. Smith, S. Lewis, T. Hewson Bache.

Place of meeting for the session of 1856, City of Philadelphia, on the last Wednesday in May, at 11 o'clock A. M.

On motion of Dr. Morton, it was

*Resolved,* That the President elect be requested to deliver an address at the opening of the next session.

On motion of Dr. Thomas, the thanks of the Society were tendered to Dr. Gemmill for the able, dignified, and courteous manner in which he has presided over its deliberations during its present session.

On motion of Dr. Morton, the thanks of the Society were tendered to its other officers for the able and efficient manner in which they have discharged their respective duties.

On motion of Dr. Thomas, the thanks of the Society were directed to be presented to the Commissioners of Blair County, for the use of the County Court House for its present session.

On motion of Dr. Biddle, the thanks of the Society were directed to be presented to the Board of Directors of the Pennsylvania Railroad Company, for the facilities afforded by them in the conveyance of the Delegates to the present session.

On motion of Dr. H. Carpenter, the thanks of the Society were tendered to the members of the profession of Blair County, for the courtesies extended by them to the Delegates in attendance at its present session.

Adjourned.

D. Francis Condie,<br>Henry Carpenter,<br>*Secretaries.*

